# Biomolecular histology as a novel proxy for ancient DNA and protein sequence preservation

**DOI:** 10.1002/ece3.9518

**Published:** 2022-12-12

**Authors:** Landon A. Anderson

**Affiliations:** ^1^ Department of Biology North Carolina State University Raleigh North Carolina USA

**Keywords:** biomolecular histology, collagen, diagenesis, fossil, molecular sequence preservation, subfossil

## Abstract

Researchers' ability to accurately screen fossil and subfossil specimens for preservation of DNA and protein sequences remains limited. Thermal exposure and geologic age are usable proxies for sequence preservation on a broad scale but are of nominal use for specimens of similar depositional environments. Cell and tissue biomolecular histology is thus proposed as a novel proxy for determining sequence preservation potential of ancient specimens with improved accuracy. Biomolecular histology as a proxy is hypothesized to elucidate why fossils/subfossils of some depositional environments preserve sequences while others do not and to facilitate selection of ancient specimens for use in molecular studies.

## Graphical abstract

Researchers' ability to accurately screen fossil and subfossil specimens for preservation of DNA and protein sequences remains limited. Cell and tissue biomolecular histology is thus proposed as a novel proxy for determining sequence preservation potential of ancient specimens with improved accuracy. Biomolecular histology as a proxy is hypothesized to elucidate why fossils/subfossils of some depositional environments preserve sequences while others do not and to facilitate selection of ancient specimens for use in molecular studies.

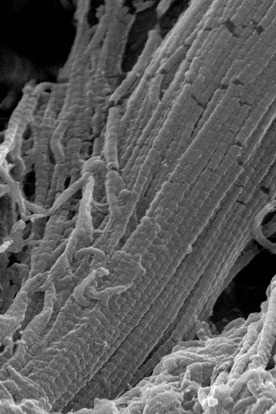

## 1 INTRODUCTION

Reports on the successful recovery of biomolecules from vertebrate fossils and subfossils (“subfossil” defined as being not fully fossilized) have increased exponentially over the prior 2 decades. Evidence supports the persistence of DNA sequences past 1 Ma (van der Valk et al., 2021), and protein sequences have been reported to preserve into the Pliocene epoch (~3.5 Ma) with minimal controversy (Buckley et al., 2019; Demarchi et al., 2016; Rybczynski et al., 2013). Predictive and empirical studies have established that the potential of sequence data to persist into deep time depends substantially on thermal setting (Demarchi et al., 2016; Hofreiter et al., 2015; Kehlmaier et al., 2017; Kendall et al., 2018; Kistler et al., 2017; Welker et al., 2019) as well as the type of biological material examined; bone, dentine, enamel, or eggshell (Demarchi et al., 2016; Kendall et al., 2018; Wadsworth & Buckley, 2014; Welker et al., 2019). These advances in DNA and protein sequence recovery have expanded the opportunity for paleoecological and paleoenvironmental studies to be conducted over a broader range of taxa and geological timepoints. Data reported from such studies are used to inform on and set conservation policy for extant wildlife populations that are of commercial interest, at risk of extinction, potentially invasive, at risk of low genetic diversity, etc. (Alter et al., 2012; Der Sarkissian et al., 2020; Jensen et al., 2022; Leonard, 2008).

Despite these advances, the ability to predict which ancient specimens are likely to preserve molecular sequences still remains limited. A prevailing view within the primary literature is that specimens exposed to prolonged, elevated thermal conditions are less likely to preserve proteins and DNA (Demarchi et al., 2016; Hofreiter et al., 2015; Wadsworth et al., 2017; Welker et al., 2019). As an example, permafrost settings and late Pleistocene geologic timepoints have been shown favorable for the preservation of molecular sequences (Hofreiter et al., 2015; Letts & Shapiro, 2012; Ngatia et al., 2019; Wadsworth & Buckley, 2014; Welker et al., 2019). Such findings have supported the use of specimen thermal history and geologic age as proxies for molecular sequence preservation (Demarchi et al., 2016; Hofreiter et al., 2015; Wadsworth et al., 2017; Welker et al., 2019). However, this observation only holds in a general sense and to a relative degree. Other variables affecting protein and DNA preservation include sediment composition (Briggs, 2003; Collins et al., 1995, 2002; Gupta, 2014; Kendall et al., 2018; Lindahl, 1993; Schweitzer et al., 2014, 2019), moisture content (Briggs, 2003; Collins et al., 2002; Gupta, 2014; Hedges & Millard, 1995; Kendall et al., 2018; Lennartz et al., 2020; Lindahl, 1993; Nielsen‐Marsh et al., 2000; Schweitzer et al., 2019; Trueman et al., 2004), and oxygen content (Briggs, 2003; Collins et al., 2002; Gupta, 2014; Kendall et al., 2018; Lennartz et al., 2020; Lindahl, 1993; Schweitzer et al., 2019; Wiemann et al., 2018, 2020), among others. Consequently, multiple studies have reported differing degrees of sequence preservation even for specimens sharing similar thermal histories and/or geologic ages (Fortes et al., 2016; Hill & Schweitzer, 1999; Letts & Shapiro, 2012; Wadsworth et al., 2017; Wadsworth & Buckley, 2014). In such situations, thermal setting and geologic age are rendered marginally effective as proxies. Indeed, the complexity of variables influencing molecular sequence preservation in vertebrate remains means that any single variable selected for use as a proxy for sequence preservation will inevitably have substantial limitations.

A potential solution to these limitations is to directly examine the biomolecular histology of preserved subfossil/fossil tissues and cells. The term “molecular histology” is defined by Campbell and Pignatelli (2002) as “an explanation of the morphological characteristics of a tissue in terms of the molecules present and the functional interactions between them” (Campbell & Pignatelli, 2002). Herein, the term “biomolecular histology” is given an equivalent definition, except that it refers strictly to tissue portions consisting of biomolecules. Regarding bone, for example, biomolecular histology would refer to collagenous matrix, osteocyte cells, blood vessels, and so on but would exclude the biogenic apatite portion of bone. Biomolecular histology is the interface of how an organism's biomolecular makeup manifests morphologically as cells and tissues, and variables including thermal history, geologic age, sediment composition, and others all directly affect how this biomolecular makeup preserves (Briggs, 2003; Briggs et al., 2000; Gupta, 2014). Thus, the preserved state of a fossil/subfossil's biomolecular histology (which includes molecular sequences), is representative of the combined effect of these diagenetic variables upon its molecular sequences. Substantial precedence exists in the scientific literature for the preservation of remnant cells and tissues within ancient vertebrate specimens (Armitage & Anderson, 2013; Boatman et al., 2019; Boskovic et al., 2021; Cadena, 2016, 2020; Lindgren et al., 2011, 2015, 2017, 2018; Schweitzer et al., 2005, 2009; Schweitzer, Wittmeyer, & Horner, 2007; Surmik et al., 2016; Wiemann et al., 2018). Data characterizing the biomolecular histology of such remains are herein hypothesized to be usable as a novel proxy for molecular sequence preservation. Correlating biomolecular histology with degree of sequence preservation across specimens spanning the fossil record would advance the use of such a proxy.

Testing this hypothesis can be accomplished using a suite of molecular techniques capable of characterizing both the morphological (Armitage & Anderson, 2013; Boatman et al., 2019; Lindgren et al., 2015; Schweitzer et al., 2005, 2013; Schweitzer, Wittmeyer, & Horner, 2007) and chemical (Lindgren et al., 2015, 2018; Long et al., 2018; Surmik et al., 2016; Thiel et al., 2014; Wiemann et al., 2018, 2020) aspects of biomolecular histology. Such data can then be used to identify connections between a specimen's observed biomolecular histology and its degree of molecular sequence preservation. Additionally, changes in fossil/subfossil biomolecular histology can be tracked across various geologic ages and depositional environments to reveal potential insights as to the role diagenetic variables play in sequence preservation. Ultimately, the study of fossil and subfossil biomolecular histology has the potential to introduce novel and practical methods for screening whether a given specimen should be selected for molecular sequencing, and to increase understanding of how geologic age, thermal history, and other diagenetic variables influence sequence preservation.

## 2 THE NEED FOR NEW PROXIES OF ANCIENT SEQUENCE PRESERVATION

The number of specimens reported to preserve molecular sequences decreases substantially beyond geologic ages of ~0.13–0.24 and ~0.8–1.0 Ma for DNA and proteins, respectively (Buckley et al., 2011; Froese et al., 2017; Lindqvist et al., 2010; Meyer et al., 2017; Mitchell & Rawlence, 2021; Wadsworth & Buckley, 2014; Welker et al., 2019). This is excluding specimens of permafrost settings and some cave deposits as these settings often confer exceptional preservation potential for molecular sequences (Dabney et al., 2013; Meyer et al., 2014, 2016; Ngatia et al., 2019; Orlando et al., 2013; van der Valk et al., 2021; Welker et al., 2019). The decrease in reported sequences from specimens exceeding these timepoints suggests substantial diagenetic alteration occurs to fossil/subfossil biomolecules over these timeframes. The extent of this diagenetic alteration is such that in many cases molecular sequences are degraded beyond the limit of detection of commonly used sequencing protocols. Still, molecular sequences, particularly protein sequences, have been reported from a few noncave/permafrost specimens with geologic ages exceeding these thresholds (Asara et al., 2007; Buckley et al., 2019; Cappellini et al., 2019; Cleland et al., 2015; Demarchi et al., 2016; Rybczynski et al., 2013; Schroeter et al., 2017; Schweitzer et al., 2009). A study on a Pliocene camel tibia from Ellesmere Island, Yukon, Canada, for example, managed to recover type‐1 collagen peptides (Buckley et al., 2019; Rybczynski et al., 2013); in addition to two Mesozoic dinosaur specimens (Asara et al., 2007; Cleland et al., 2015; Schroeter et al., 2017; Schweitzer et al., 2009), these are the only pre‐Pleistocene bones currently reported to harbor sequenceable proteins. Two other camels from Miocene and Pliocene formations of Nebraska were analyzed in the Ellesmere Island tibia study yet failed to yield detectable peptide sequences (Buckley et al., 2019; Rybczynski et al., 2013). This begs the question of why some specimens like the exceptional Ellesmere Island tibia preserve protein and/or DNA sequence information while many other pre/early and even mid‐Pleistocene specimens do not.

The prevailing view in the paleogenomic and paleoproteomic literature would be that the greater thermal exposure of the temperate Nebraska specimens facilitated protein degradation relative to the Ellesmere Island tibia (Demarchi et al., 2016; Hofreiter et al., 2015; Kistler et al., 2017; Wadsworth et al., 2017; Welker et al., 2019). A warmer thermal setting accelerates the rate of diagenetic reactions affecting biomolecular histology, including molecular sequences (Demarchi et al., 2016; Kistler et al., 2017; Ramsøe et al., 2021). Advanced geologic age expands the temporal period over which these reactions have to progress and accumulate (Kistler et al., 2017; Lindahl, 1993; Ramsøe et al., 2021). Hence, a lower geologic age along with a cooler thermal setting is hypothesized to inhibit the extent of such diagenetic reactions and limit molecular sequence degradation. This, and the degree materials such as bone, dentine, enamel, eggshell, and others resist degradation (Demarchi et al., 2016; Wadsworth & Buckley, 2014; Welker et al., 2019) are often cited as key variables explaining examples of exceptional sequence preservation.

Indeed, a fossil or subfossil's thermal setting/history and geological age are generally used as proxies for predicting sequence preservation potential (Demarchi et al., 2016; Hofreiter et al., 2015; Kistler et al., 2017; Wadsworth et al., 2017; Welker et al., 2019). However, even ancient specimens from similar timepoints and depositional environments are known to display great variation in sequence preservation (Fortes et al., 2016; Hill & Schweitzer, 1999; Kistler et al., 2017; Letts & Shapiro, 2012; Mackie et al., 2017; Presslee et al., 2019; Wadsworth & Buckley, 2014; Wadsworth et al., 2017). In a study of 118 *Xenarthrans* from temperate to tropical locales, six specimens from the Santa Clara formation (~8.5–128 Ka) of Camet Norte, Buenos Aires, Argentina, were analyzed. Of these, two specimens out of six demonstrated substantial evidence of protein preservation (Presslee et al., 2019). In this case, geologic age and thermal setting would be rendered relatively inaccurate as proxies since all specimens came from the same formation and would be expected to share a similar thermal history, yet not all preserve sequence information to a similar degree. Furthermore, a 2017 study by Mackie et al. examined the dental calculus of 21 Roman‐era *Homo sapiens* specimens from three European burial sites using LC–MS/MS sequencing. Reported sequence preservation varied widely between specimens and was unattributable to any specific variables (Mackie et al., 2017). These differences in preservation likely result from a combination of other variables including differences in composition (Briggs, 2003; Collins et al., 1995, 2002; Gupta, 2014; Kendall et al., 2018; Lindahl, 1993; Schweitzer et al., 2014, 2019), moisture content (Briggs, 2003; Collins et al., 2002; Gupta, 2014; Kendall et al., 2018; Lennartz et al., 2020; Lindahl, 1993; Nielsen‐Marsh et al., 2000; Schweitzer et al., 2019; Trueman et al., 2004), and oxygen content (Briggs, 2003; Collins et al., 2002; Gupta, 2014; Kendall et al., 2018; Lennartz et al., 2020; Lindahl, 1993; Schweitzer et al., 2019; Wiemann et al., 2018, 2020) of burial sediments, among others. The complex range of variables potentially affecting sequence preservation supports that factors beyond geologic age and thermal history are responsible for specimens demonstrating exceptional sequence preservation. This limits the usefulness of any single diagenetic variable, such as geologic age or thermal history, as a proxy for DNA and protein sequence preservation.

A proposed solution to this limitation is to directly use fossil/subfossil biomolecular histology as a proxy for molecular sequence preservation. Biomolecular histology is the underlying basis for why diagenetic variables such as thermal history and geologic age can be used as proxies, in any capacity, for predicting sequence preservation. The cumulative effects of diagenetic variables are reflected in the preservational condition of a fossil or subfossil's biomolecular histology (Briggs, 2003; Briggs et al., 2000; Gupta, 2014). Directly studying biomolecular histology and correlating it with degree of sequence preservation bypasses the need to study any one of these variables individually. Thus, biomolecular histology is hypothesized to be usable as an accurate proxy for molecular sequence preservation. Yet, little empirical research exists to this point that has observed how biomolecular histology of fossil and subfossil specimens varies with degree of sequence preservation.

## 3 BIOMOLECULAR HISTOLOGY AS A NOVEL PROXY FOR ANCIENT SEQUENCE PRESERVATION

A biomolecular histological approach directly examines the interface between tissue morphology and constituent biomolecules. One approach to studying biomolecular histology is to analyze biomolecule morphology. An example is electron microscope imaging of the ~67‐nm banded fibrils that are the direct manifestation of type‐1 collagen peptide sequences (Boatman et al., 2019; Gottardi et al., 2016; Lin et al., 1993; Rabotyagova et al., 2008; Tzaphlidou, 2005). Another example is the microscopic imaging of cellular membranes, which are primarily the manifestation of phospholipid bilayers with associated proteins and sterols (Lamparter & Galic, 2020). In both cases, the direct morphological manifestation of biomolecules is being examined. In contrast, prior studies correlating fossil/subfossil tissue histology with molecular sequence preservation have generally done so using petrographic thin sections of mineralized tissue, such as whole bone (Collins et al., 2002; Hollund et al., 2017; Kontopoulos et al., 2019; van der Sluis et al., 2014). The presence of both abundant biogenic and/or exogenous minerals hinders the observation of biomolecule morphology (Armitage & Anderson, 2013; Collins et al., 2002; Kontopoulos et al., 2019; van der Sluis et al., 2014), obscuring features of collagenous matrix, cellular membranes, vascular tissue, and other such structures. With light microscopy, such structures often may not even be observable in petrographic thin sections. A biomolecular histological approach would instead isolate biomolecular tissue portions from surrounding biogenic minerals. For example, in the case of bone, collagenous matrix, blood vessels, osteocyte cells, and so on would be individually isolated from surrounding bioapatite mineral, potentially via incubation in dilute acid (Armitage & Anderson, 2013; Lindgren et al., 2018; Schweitzer, Wittmeyer, & Horner, 2007; Schweitzer et al., 2013; Surmik et al., 2016). These biomolecular tissue portions would then be observable unhindered by biogenic minerals. Observations of properties of these biomolecular structures such as flexibility, robustness, and color, among others, can then be readily made using light microscopy, and surface structure is less obscured during electron microscope imaging (Lindgren et al., 2011; Schweitzer, Wittmeyer, & Horner, 2007; Schweitzer et al., 2005; Surmik et al., 2016). The absence of ~67‐nm banding in type‐1 collagen fibrils, for example, of demineralized collagenous matrix directly evidences a substantial degree of collagen sequence degradation (Carrilho et al., 2009; Hashimoto et al., 2003; Rabotyagova et al., 2008). Such changes to the fibrils indicate the type‐1 collagen peptide sequences have shifted in structure at the molecular level, which corresponds to degradation (Rabotyagova et al., 2008). The degree of change is hypothesized to correspond to degree of chemical sequence degradation, although, as this manuscript discusses, this has not yet been tested for ancient specimens. A petrographic thin section demonstrating somewhat degraded bone histology does not likewise necessarily indicate poorly preserved collagenous matrix/peptides (although it certainly may be suggestive). Such degradation could be more so due to alteration of the biogenic apatite or perhaps the incorporation of exogenous minerals or other contaminants. Biomolecular histology is proposed as a more precise/higher resolution proxy of molecular preservation relative to the historical methods that analyze whole mineralized hard tissue.

Regarding the study of biomolecular histology, an alternative to examining biomolecule morphology, as described above, is to instead chemically localize/map biomolecular signal to histological structure. For example, time‐of‐flight secondary ionization mass spectrometry (ToF‐SIMS) can map biomolecular signals across histological structure surface. Ionized fatty acids and phospholipids, along with a variety of ionic fragments (including those of proteins), can be localized/mapped across cellular membranes (Sodhi, 2004; Thiel & Sjövall, 2011; Touboul & Brunelle, 2016). Historically, studies correlating biomolecular signal with molecular sequence preservation have done so using homogenized samples (demineralized or powdered) (Campos et al., 2012; Hollund et al., 2017; Kontopoulos et al., 2018, 2019, 2020; Leskovar et al., 2020; van der Sluis et al., 2014). In Kontopoulos et al. (2019, 2020), organics are directly examined using FT‐IR to obtain amide/phosphate signal ratios for specimens. However, these ratios are obtained from homogenously ground bone which precludes localizing the biomolecular signal to its histological source (Kontopoulos et al., 2019, 2020). A biomolecular histological approach would instead use imaging FT‐IR to localize chemical signal to specific histological structures (Pan et al., 2019), such as collagen matrix fibrils or cellular membranes. This approach improves confidence that the chemical signal is indeed endogenous, and that it originates from the target structure; for example, if FT‐IR data for collagen protein specifically are desired, imaging FT‐IR allows for structure consistent with collagen fibrils to be directly analyzed, as opposed to analysis of homogenized samples. This reduces the likelihood of potential contamination and allows for a more direct and replicable comparison of biomolecular structure, such as collagenous matrix or cellular membranes, across fossil/subfossil specimens. Again, biomolecular histology is here proposed as a novel proxy for sequence preservation that is more precise and higher resolution relative to prior methodologies such as sampling of homogenized tissue.

In general, the use of biomolecular histology as a proxy is less assuming than historical methods. Stable isotope, spectroscopic, and other analyses applied to homogenized samples generally assume (to an extent) that chemical signals arise from endogenous biomolecules (Campos et al., 2012; Chadefaux et al., 2009; Hollund et al., 2017; Kontopoulos et al., 2018, 2020; Lebon et al., 2016; van der Sluis et al., 2014); in the case of homogenized bone, this signal is generally attributed to the highly abundant collagenous matrix (Hollund et al., 2017; Kontopoulos et al., 2019, 2020; Leskovar et al., 2020). For petrographic thin sections and other methods utilizing mineralized tissue histology, histological preservation of mineralized tissue is generally assumed to correlate, to an extent, with degree of biomolecular preservation (Collins et al., 2002; Kontopoulos et al., 2019; Nielsen‐Marsh et al., 2007). For younger subfossil specimens that are *relatively* unaltered, these certainly may be reasonable assumptions. When dealing with increasingly ancient and/or diagenetically altered remains, however, the potential for unknown variables affecting specimen diagenesis increases (Alleon et al., 2021; Buckley et al., 2017; Hollund et al., 2017; Schweitzer et al., 2002). Furthermore, claims of endogenous organics are especially scrutinized for increasingly ancient specimens, or those likely subjected to extensive diagenetic alteration, such as from tropical and subtropical thermal settings. In such cases, assuming the source of biomolecular signals is especially risky, and such claims are likely to be challenged (Alleon et al., 2021; Buckley et al., 2017; Kaye et al., 2008; Saitta et al., 2019). Rather than use analyses of complex samples, such as, for example, whole bone sections or homogenously ground bone, specific changes to the collagen protein fibrils of bone could instead be observed. As mentioned earlier, such observations include microscopic imaging of the ~67‐nm banded fibril structure formed by type‐1 collagen peptide sequences (Boatman et al., 2019; Gottardi et al., 2016; Lin et al., 1993; Rabotyagova et al., 2008; Tzaphlidou, 2005), or the mapping and localization of biochemical signal to the collagen fibrils. This direct observation of the type‐1 collagen fibrils themselves limits the potential for unknown variables to impact the use of such biomolecular histological data as a proxy. The result is a potentially higher resolution, more precise proxy for ancient DNA and protein sequence preservation.

**FIGURE 1:**
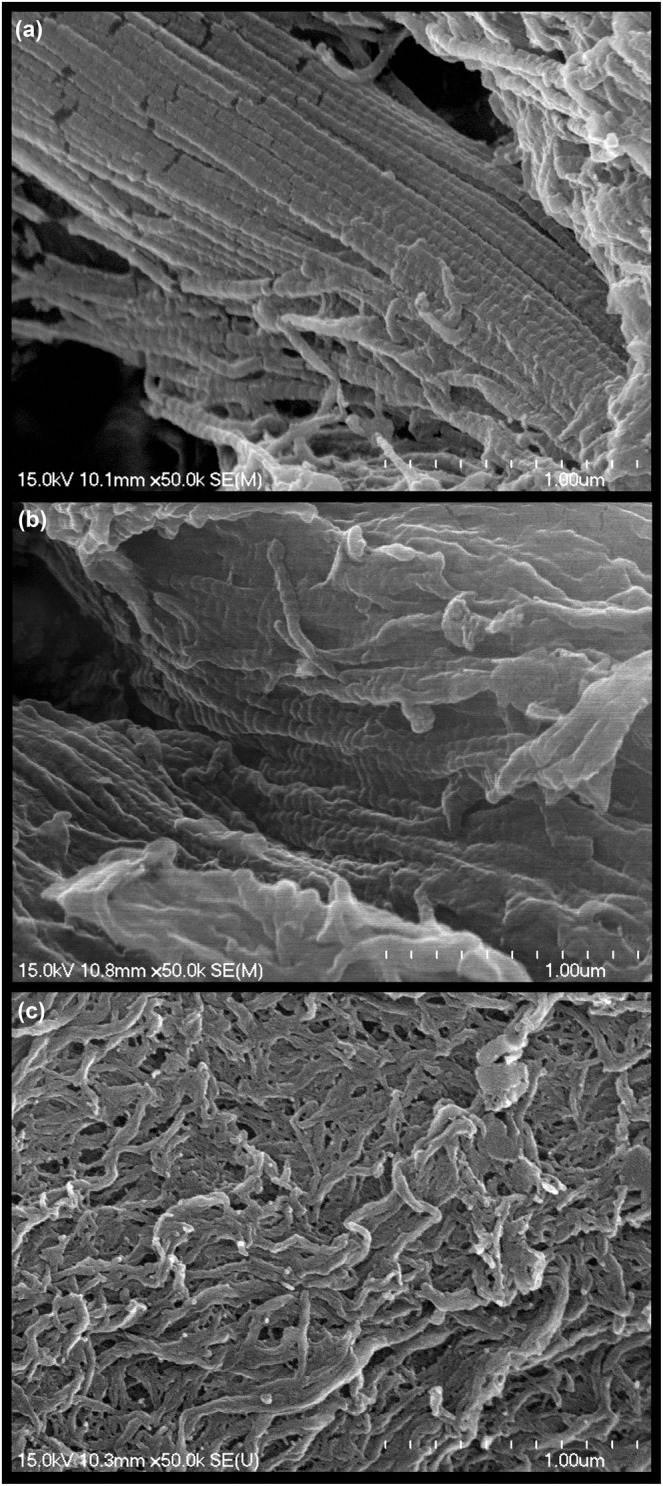
Scanning electron microscope images of “type‐1 bone collagen” demineralized bone matrix fibrils. (a) Fibrils from the *B. taurus* extant long bone control. Prominent banding (~67‐nm) is present that is characteristic of type‐1 collagen protein fibrils (Boatman et al., 2019; Gottardi et al., 2016; Lin et al., 1993; Rabotyagova et al., 2008; Tzaphlidou, 2005). (b) Permafrost YG 610.2397 *M. primigenius* demineralized bone matrix fibrils. An ~67‐nm banding pattern on the fibrils is also observed but is somewhat less distinct in comparison to that of the extant *B. taurus* specimen. (c) Observed fibril structures in the temperate MOR 91.72 *M. columbi* specimen. Fibril banding is generally absent, suggesting the original chemical state of the type‐1 collagen fibrils/sequences is substantially altered. Methods: An ~200–300 mg fragment of each specimen was demineralized in EDTA (0.5 M, pH 8.0) for ~1–3 days, fixed for 1 h in 2.5% glutaraldehyde (multiple washes in phosphate‐buffered saline were performed before and after fixation to remove glutaraldehyde), and dehydrated in a graded series of ethanol incubations (1 h at 50%, 1 h 70%, 1 h 95%, 3x 1 h 100% ethanol). Post dehydration, specimens were critical point dried (Tousimis Autosamdri‐931), sputter coated (Cressington 108 Auto) with ~70 angstroms of palladium gold metal, and imaged with a Hitachi S‐4700 Cold Cathode Field Emission Scanning Electron Microscope. All images were taken at 50,000x magnification and with an accelerating voltage of 15.0 kV. Resultant electron microscope images were processed in Adobe Photoshop 2021, using the Levels tool, with a histogram stretch, followed by a gamma adjustment, followed by a second histogram stretch. Sample preparation for ancient specimens was done in a dedicated “ancient” clean lab separate from the extant control, and was performed wearing gloves, a laboratory coat, a surgical mask, and a bouffant cap. Sampling of ancient specimens was done using a hammer and chisel sterilized with 10% bleach followed by 70% ethanol. Laboratory surfaces near preparatory areas for ancient samples were also sterilized with 10% bleach followed by 70% ethanol, and all glassware and consumables were autoclaved prior to use. Solutions for ancient specimens were vacuum filtered (0.220 microns) prior to use in preparation protocol. For critical point drying, sputter coating, and imaging, samples were transported from the clean lab to the CHANL core facility at the University of North Carolina at Chapel Hill.

A study by Cappellini et al. (2012) (Cappellini et al., 2012) used LC–MS/MS to recover peptide sequences from bones of a permafrost *Mammuthus primigenius* specimen (~43 Ka, Yakutia, Russia) and two temperate *Mammuthus columbi* specimens (~11 Ka, Colorado, United States; ~18 Ka, Nebraska, United States). For the *M. primigenius* specimen, 1139 unique peptides were recovered matching 269 different proteins with at least one unique peptide sequence; 126 proteins were reported to match at least 2 unique peptide sequences for this specimen. In contrast, for the two temperate *M. columbi* specimens, 342 and 243 unique peptides were recovered matching 35 and 19 different proteins from the Colorado and Nebraska *M. columbi* specimens, respectively. This corresponds to a > 85% decrease in the number of unique proteins identified between the permafrost *M. primigenius* and the two temperate *M. columbi*. As a preliminary investigation, to demonstrate the concept of correlating biomolecular histology to degree of sequence preservation, bone samples relatively comparable (similar geologic age and thermal history) to those sequenced by Cappellini et al. (2012) were herein obtained, along with an extant control. Scanning electron microscope images of demineralized bone matrix were obtained (Figure 1) from a *Bos taurus* long bone (extant, fresh, and frozen only once, purchased from Whole Foods Market), a Beringian permafrost *Mammuthus primigenius* innominate fragment (Pleistocene, Little Blanche Creek, Yukon territory, Canada, YG 610.2397), and a temperate *Mammuthus columbi* femur specimen (~14–15 Ka in calibrated years, ~12.5 Ka in radiocarbon years, Lindsay/Deer Creek, Montana, United States, MOR 91.72).

The observable differences in preservation across the images in Figure 1 are a preliminary example of how biomolecular histology can potentially be used to screen specimens for degree of DNA and protein sequence preservation. The extant *B. taurus* fibrils (Figure 1a) show the prominent ~67‐nm banding pattern characteristic of type‐1 bone collagen (Boatman et al., 2019; Gottardi et al., 2016; Lin et al., 1993; Rabotyagova et al., 2008; Tzaphlidou, 2005). The permafrost YG 610.2397 *M. primigenius* fibrils (Figure 1b) also show this banding, but it is potentially less distinct/ordered. In contrast, the temperate *M. columbi* MOR 91.72 fibrils (Figure 1c) show a general absence of this banding pattern, suggesting degradation of type‐1 collagen protein sequences. This would be consistent with MOR 91.72 being recovered from a temperate region, unlike the permafrost YG 610.2397 innominate fragment. These differences are consistent with the difference in number of proteins recovered between the permafrost (269) and temperate specimens (35 and 19) of Cappellini et al. (2012) (Cappellini et al., 2012). While this comparison is strictly suggestive because it is between different, although relatively comparable, specimens, it demonstrates the potential of biomolecular histology as a proxy of ancient DNA and protein sequence preservation. Because the collagen fibrils shown in Figure 1 are the direct morphological manifestation of type‐1 collagen sequences, any observed degradation to this morphology is hypothesized to correspond to type‐1 collagen sequence degradation and with further testing may be usable as a proxy.

## 4 LITERATURE EXAMPLES HIGHLIGHTING THE POTENTIAL OF BIOMOLECULAR HISTOLOGY AS A PROXY

A few previous studies have examined fossil/subfossil biomolecular histology in a manner that can be linked to preservation potential for molecular sequences. A discussion of some findings relevant to the correlation of biomolecular histology with degree of sequence preservation follows.

A 2007 study by Schweitzer et al. used light and electron microscopy to survey the biomolecular histology of bone specimens ranging from modern day through to the Triassic (Schweitzer, Wittmeyer, & Horner, 2007). The study reported that the biomolecular histology (especially the “collagenous” matrix) of specimens with dates exceeding ~100–600 Ka was substantially altered relative to specimens of younger timepoints. Light microscopy was herein used to replicate and reevaluate reported data for three of the 2007 study specimens (data not shown), the *M. columbi* femur (MOR 91.72, ~14–15 Ka in calibrated years, ~12.5 Ka in radiocarbon years, Lindsay/Deer Creek, Montana, United States; Hill & Davis, 1998; Hill & Schweitzer, 1999), the *M. columbi* skull (MOR 604, ~100–600 Ka, Doeden gravel beds, Montana, United States; Hill & Schweitzer, 1999; Schweitzer et al., 2002; Wilson & Hill, 2000), and the *M. pacificus* skull (MOR 605, ~100–600 Ka, Doeden gravel beds, Montana, United States, species recently reassigned from *M. americanum*; Asara et al., 2007; McDonald et al., 2020; Wilson & Hill, 2000). This was done according to the same demineralization protocol reported by the 2007 study (Schweitzer, Wittmeyer, & Horner, 2007).

“Collagenous” matrix of the mid‐Pleistocene MOR 604 and MOR 605 specimens was highly fragmented and brittle, supporting substantial degradation. Histological structures resembling blood vessels readily broke free of the degraded matrix and were easily isolated. Both specimens exhibited evidence of exogenous, orange‐brown mineralization across portions of structure surfaces even after hydroxyapatite was removed via acid demineralization. In contrast, the late Pleistocene MOR 91.72, also from the temperate region of Montana, U.S.A. (Schweitzer, Wittmeyer, & Horner, 2007), preserved a structured, *relatively* intact collagenous matrix. Emphasis is placed on the term *relatively*, as Figure 1 demonstrates the collagenous matrix of MOR 91.72 itself is still somewhat degraded relative to extant specimens. No evidence for exogenous mineralization of MOR 91.72 was detected with light microscopy.

Matrix from MOR 91.72 was more consistent in morphology and robustness with past reports on extant collagenous matrix (Schweitzer et al., 2005, 2013) than what was observed for matrix of MOR 604 and MOR 605. “Collagenous” matrix of MOR 604 and MOR 605 was closer in morphology to what has previously been reported for Mesozoic dinosaurs (Schweitzer et al., 2005, 2009; Schweitzer, Wittmeyer, & Horner, 2007) and early–mid Cenozoic organisms (Cadena, 2016, 2020). The stark difference in these observations supports a disparity in degree of type‐1 collagen preservation between these specimens, which is predicted to affect potential sequencing analyses. Prior studies have reported type‐1 collagen sequences from MOR 604 (Schweitzer et al., 2002) and MOR 605 (Asara et al., 2007). MOR 91.72 however has not previously been sequenced and a direct comparison regarding degree of type‐1 collagen sequence preservation is not currently possible. Further, MOR 91.72, MOR 604, and MOR 605 were all recovered from the same geographic region, albeit different burial sites (Schweitzer, Wittmeyer, & Horner, 2007). This supports the observed dichotomy in “collagenous” matrix preservation is thus likely less dependent on thermal setting.

Another study that analyzed biomolecular histology to a limited extent is that of the Pliocene Ellesmere Island camel tibia (Rybczynski et al., 2013). A cross‐section of a vascular canal within the tibia was elementally mapped using energy dispersive X‐ray spectroscopy (EDS). The analysis demonstrated that elements consistent with iron oxyhydroxides and barium sulfates colocalized to the vascular canal. The presence of such exogenous minerals supports that this tibia had undergone substantial chemical alteration. Both mineral precipitants are consistent with observations from older tertiary (Boskovic et al., 2021; Cadena, 2016, 2020) and even Mesozoic specimens (Armitage & Anderson, 2013; Boatman et al., 2019; Schweitzer et al., 2013, 2014; Surmik et al., 2016), and their presence likely precludes it from being considered a “subfossil.” Despite the apparent chemical alteration to its biomolecular histology, the tibia still preserved collagen sequences identifiable via mass spectrometry (Rybczynski et al., 2013).

Samples from the Pliocene tibia were not demineralized and examined with light microscopy within the study (Rybczynski et al., 2013), however, thus precluding a direct comparison against observations for the MOR 91.72, MOR 604, and MOR 605 “collagenous” matrix morphology. The substantial mineralization detected by the EDS analysis is consistent with observations of mineralized histological structures within MOR 604 and MOR 605 (Schweitzer, Wittmeyer, & Horner, 2007). This supports a hypothesis that any “collagenous” matrix the Ellesmere Island tibia preserves is likely highly degraded morphologically, in a manner consistent with MOR 604 and MOR 605 (Schweitzer, Wittmeyer, & Horner, 2007) as well as previous reports for Mesozoic dinosaurs (Schweitzer, Wittmeyer, & Horner, 2007) and pre‐Pliocene Cenozoic (Boskovic et al., 2021; Cadena, 2016, 2020) specimens.

Data from the two studies above already enables some predictions to be made regarding the relationship of the specimens' underlying biomolecular histology with degree of sequence preservation, and even some diagenetic variables. The dichotomy in biomolecular histology between extant specimens along with MOR 91.72 when compared against MOR 604, MOR 605, the Pliocene camel tibia, and Mesozoic dinosaurs is to this point a largely unexplored finding. Few, if any, studies have directly explored how these differences in demineralized “collagenous” matrix histology manifest in degree of recovered sequence data.

Based on the discussion above, bone specimens preserving ancient DNA are herein hypothesized to possess an intact, robust collagenous matrix somewhat similar to that of MOR 91.72. If sequence‐able DNA is present, collagenous matrix would also still be expected to be relatively intact. In contrast, bone specimens with a brittle, easily fragmented “collagenous” matrix like that of MOR 604 and MOR 605 are predicted to preserve, at most, remnant peptide sequences. If the collagenous matrix has degraded to the point it has lost much of its structural integrity, the preservation of sequenceable DNA is not expected (Briggs et al., 2000; Lindahl, 1993; Wang et al., 2012). Further, this agrees with the trend of sequenceable DNA being rarely reported from specimens exceeding 0.13–0.24 Ma in geologic age (excluding cave and permafrost deposits) (Buckley et al., 2011; Froese et al., 2017; Lindqvist et al., 2010; Meyer et al., 2017; Mitchell & Rawlence, 2021; Wadsworth & Buckley, 2014; Welker et al., 2019) as MOR 604 and MOR 605 are both assigned constrained dates of ~100–600 Ka (Hill & Schweitzer, 1999; McDonald et al., 2020; Wilson & Hill, 2000).

If such a hypothesis were supported, practical methods such as electron and even light microscopy may be capable of screening fossil/subfossil specimens for sequence preservation with high precision. However, the limited extent of the data that has been reported for ancient vertebrate biomolecular histology severely limits the conclusions that can be drawn regarding these relationships. This epitomizes the need emphasized by this review for extensive study of fossil/sub‐fossil vertebrate biomolecular histology.

## 5 METHODS FOR STUDYING FOSSIL/SUBFOSSIL BIOMOLECULAR HISTOLOGY

Examination of fossil/subfossil biomolecular histology is proposed for empirically studying how the cumulative effect of diagenetic variables upon a specimen's biomolecular histology correlates with degree of sequence preservation. Vertebrate elements with the highest potential for molecular sequence preservation include tooth enamel and dentine, bone, and eggshell (Demarchi et al., 2016; Wang et al., 2012; Welker et al., 2019). Of these, bone by far is the most widely characterized within ancient specimens as to its nonmineral histological structures. Numerous studies have reported histological structures morphologically and chemically consistent with biological cells, vascular tissue, and “collagenous” matrix preserved within Cenozoic and Mesozoic bones (Armitage & Anderson, 2013; Boatman et al., 2019; Boskovic et al., 2021; Cadena, 2016, 2020; Lindgren et al., 2011, 2015, 2017, 2018; Schweitzer et al., 2005, 2009; Schweitzer, Wittmeyer, & Horner, 2007; Surmik et al., 2016; Wiemann et al., 2018). In particular, the organic portion of extant collagenous bone matrix is comprised of ~90% type‐1 bone collagen (Boatman et al., 2019; Wang et al., 2012). This high proportion of a single, specific molecule is practical for comparison against purified collagen standards, extant controls, and across various ancient specimens.

The above histological structures are generally isolated via demineralization using a dilute acid (Lindgren et al., 2018; Schweitzer, Wittmeyer, & Horner, 2007; Surmik et al., 2016); this allows their biomolecular histology to be investigated using a suite of molecular methods. Characterization of morphology for these structures has historically been accomplished using a combination of light microscopy and both of transmission and scanning electron microscopy (Armitage & Anderson, 2013; Lindgren et al., 2018; Schweitzer et al., 2005, 2013; Schweitzer, Wittmeyer, & Horner, 2007; Surmik et al., 2016). Light microscopy is a practical method to rapidly screen specimens for the preservation of histological structures. The use of both transmission and scanning electron microscopy together is particularly advantageous. While both offer nanoscale optical resolution, the former images sample cross‐sections while the latter sample surface (Bozzola & Russell, 1999; Handbook of Microscopy, 2019). Both methods are also readily capable of detecting a distinct ~67‐nm banding pattern unique to collagen protein helices (Boatman et al., 2019; Gottardi et al., 2016; Lin et al., 1993; Tzaphlidou, 2005). Observation of this banding pattern indicates either the presence of a collagen helix or compounds replicating its structure.

Studying the chemical aspect of biomolecular histology generally requires localizing chemical signal to a specific histological structure. Two methods with precedence for use within molecular paleontology are ToF‐SIMS and Raman spectroscopy:

1. ToF‐SIMS rasters a micro/nanoscale‐diameter ion beam in a square, grid‐like pattern across a specimen's surface. At each point in the square analysis “grid,” the chemical content of the specimen's surface (uppermost ~1–2 nm) at that specific point is detected and recorded as a spectrum of molecular and fragment ions. A specific ion can then be plotted according to its recorded intensity at each point in the grid to form a molecular map that mirrors the area analyzed across the specimen's surface. The specific types of ions detected via this process vary depending upon specimen chemical makeup; this allows the unique histological structures of a specimen to be targeted so that chemical makeup can be connected to morphology (Sodhi, 2004; Thiel & Sjövall, 2011; Touboul & Brunelle, 2016). A few studies have employed ToF‐SIMS to analyze ancient specimens (Lindgren et al., 2012, 2015, 2017, 2018; McNamara et al., 2016; Orlando et al., 2013; Schweitzer, Suo, et al., 2007; Surmik et al., 2016). One recent publication used the method to analyze the biomolecular histology of demineralized epidermis from an exceptionally preserved Jurassic ichthyosaur (Lindgren et al., 2018). Ionic fragments consistent with peptides or related compounds, along with polyaromatic hydrocarbons, were successfully localized to the ichthyosaur epidermis. Recorded intensities for polyaromatic hydrocarbon and peptide‐related ion fragments (such as those detected in the Jurassic ichthyosaur (Lindgren et al., 2018)) can be compared across extant and ancient histological structures. For example, elevated levels of polyaromatic related ions in one specimen relative to another would be predicted to indicate a higher degree of chemical degradation (Buseck & Beyssac, 2014; Delarue et al., 2016; Oberlin, 1984; Sjövall et al., 2021; Thiel & Sjövall, 2011). This is one potential method for evaluating changes in fossil/subfossil biomolecular histology by geologic timepoint and depositional environment.
2. Raman spectroscopy utilizes a monochromatic laser to irradiate (typically) a single point a few microns in diameter on a specimen surface (Ferraro et al., 2003; Pan et al., 2019; Smith & Dent, 2019). As the laser's photons contact the specimen surface, a small number of them are inelastically scattered by the specimen surface; that is, they either gain or lose energy after contacting the specimen surface (Raman & Krishnan, 1928; Smekal, 1923). The degree to which these photons change energy depends on the type of molecular bond vibration the photon interacted with within the specimen. Detecting the change in these photons' energies forms a spectrum revealing the types of molecular bond vibrations present where the laser contacted. This allows specific histological structures to be analyzed for the types of molecular bonds present in their chemical makeup (Ferraro et al., 2003; Hill et al., 2020; Pan et al., 2019; Smith & Dent, 2019). A recent study attempted to analyze the biomolecular histology of fossil tissues using Raman spectroscopy (Wiemann et al., 2018). However, perusal of their published findings raised questions as to whether some of their data represented true Raman signal or was an artifact of autofluorescence (Alleon et al., 2021). Raman spectroscopy with a laser wavelength below 250‐nm is a well‐established solution to eliminate autofluorescence (Abbey et al., 2017; Long et al., 2018) but has seen little use within molecular paleontology historically (Long et al., 2018). However, similar to the ion intensities with ToF‐SIMS, Raman signal intensity for specific bond vibrations can be compared across extant and ancient specimen biomolecular histology. Indeed, this method has seen substantial use historically in correlating thermal history with molecular makeup for a wide range of humics and kerogen macromolecules in petroleum and soil science (Delarue et al., 2016; Ferralis et al., 2016; Schito et al., 2017).

Data collected using these described techniques can be correlated with the degree to which molecular sequences are recoverable from fossil and subfossil specimens. Both the intensity of Raman signal for specific bond vibrations and the relative ion abundances from ToF‐SIMS can readily be compared against the degree to which a specimen preserves molecular sequence information. In the case of collagen peptides, both forms of electron microscopy can be used to evaluate the relative abundance of ~67‐nm banding present within bone matrix. This too can be compared against the degree of type‐1 collagen sequence information recoverable from a given specimen (similar to the preliminary case study described in Figure 1).

## 6 ADVANTAGES FOR SAMPLING OF ANCIENT SPECIMENS

Regarding fossil/subfossil specimen sampling, the above described methods generally function over a scale of micrometers to nanometers (Bozzola & Russell, 1999; Ferraro et al., 2003; Handbook of Microscopy, 2019; Marini et al., 2015; Pan et al., 2019; Smith & Dent, 2019; Sodhi, 2004; Thiel & Sjövall, 2011). Small samples of tens to hundreds of milligrams will suffice for any one of these molecular methods, provided care is taken during sample preparation. This limits the extent of destructive sampling necessary to study fossil/subfossil specimen biomolecular histology. Particularly, this allows for minimally destructive sampling of specimens that preserve exceptional morphology; this includes articulation, fossil organs, color, among other examples (Brown et al., 2017; Greenwalt et al., 2013; Lindgren et al., 2015, 2017, 2018; Manning et al., 2009; Yamagata et al., 2019). Examination of specimen biomolecular histology is hypothesized to yield insight into the general preservational state of such specimens at the molecular level. This would inform on whether future destructive molecular analyses, including sequencing, are justified for such morphologically exceptional specimens. If initial analysis of biomolecular histology suggests that a given “exceptional” specimen has limited potential for molecular sequence preservation, destructive sampling can be halted.

Furthermore, several recent studies have demonstrated isolated, disarticulate remains, even those stored for extended periods in museum collections, can often be used in molecular analyses in place of exceptionally preserved specimens that are more informative morphologically (Bertazzo et al., 2015; Cleland et al., 2016; Ngatia et al., 2019; Wiemann et al., 2018). The potential use of such specimens would improve stewardship of fossil and subfossil resources. Studying the biomolecular histology of such morphologically unexceptional specimens is hypothesized to further advance understanding on which geologic timepoints and depositional environments are most likely to harbor fossils/subfossils preserving ancient sequences. Advancing such knowledge, in this way, would then help limit the unnecessary sampling of more morphologically exceptional fossil/subfossil specimens that are otherwise unlikely to preserve sequenceable biomolecules at the molecular level, based on their diagenetic history.

## 7 CONCLUSION

Thermal setting and geologic age have been commonly used as proxies for predicting molecular sequence preservation potential (Demarchi et al., 2016; Hofreiter et al., 2015; Wadsworth et al., 2017; Welker et al., 2019). Late Pleistocene and Holocene specimens from cooler regions, especially permafrost deposits, have been shown to generally possess the highest preservation potential for molecular sequence information (Hofreiter et al., 2015; Letts & Shapiro, 2012; Ngatia et al., 2019; Wadsworth & Buckley, 2014; Welker et al., 2019). However, depositional environments are influenced by other variables including moisture (Briggs, 2003; Gupta, 2014; Lennartz et al., 2020; Lindahl, 1993; Schweitzer et al., 2019) and oxygen content (Briggs, 2003; Gupta, 2014; Lindahl, 1993; Schweitzer et al., 2019; Wiemann et al., 2018, 2020), ion species present, and sediment composition (Briggs, 2003; Gupta, 2014; Lindahl, 1993; Schweitzer et al., 2014, 2019). These confounding variables limit the usefulness of thermal setting and geologic age as proxies outside of a broad scale.

Direct analysis of fossil and subfossil biomolecular histology is a potential answer to this limitation. The biomolecular histology of a specimen's preserved cells and tissues reflects the cumulative effects of environmental variables upon its constituent biomolecules, including DNA and protein sequences (Briggs, 2003; Briggs et al., 2000; Gupta, 2014). Observed degradation of cell and tissue biomolecular histology is hypothesized to correlate with constituent biomolecules having undergone degradation. This agrees with the limited data in the primary literature on the correlation of biomolecular histology with sequence preservation potential (Asara et al., 2007; Rybczynski et al., 2013; Schweitzer et al., 2002; Schweitzer, Wittmeyer, & Horner, 2007). Thus, the preserved state of fossil/subfossil biomolecular histology is predicted to be an accurate proxy for molecular sequence preservation. A potential limitation to this approach is that some aspects of biomolecular histology may be beyond resolution or limit of detection for current molecular methods. However, modern molecular instrumentation regularly functions on the micro‐ and nanoscale in terms of resolution and limit of detection (Bozzola & Russell, 1999; Ferraro et al., 2003; Handbook of Microscopy, 2019; Marini et al., 2015; Pan et al., 2019; Smith & Dent, 2019; Sodhi, 2004; Thiel & Sjövall, 2011), thus minimizing this limitation as a potential obstacle. The use of fossil/subfossil biomolecular histology as a proxy for sequence preservation has potential to elucidate why ancient specimens of some formations and timepoints preserve sequences while others do not; such understanding would facilitate the selection of ancient specimens for use in future ancient DNA and paleoproteomic studies.

## AUTHOR CONTRIBUTIONS

**Landon A. Anderson:** Conceptualization (lead); formal analysis (lead); investigation (lead); methodology (lead); project administration (lead); software (lead); supervision (lead); validation (lead); visualization (lead); writing – original draft (lead); writing – review and editing (lead).

## CONFLICT OF INTEREST

There are no competing interests to declare.

## Data Availability

Data from this work is available in the Dryad public repository: https://doi.org/10.5061/dryad.8gtht76sq.
